# Physiological Adaptation of *Fenneropenaeus chinensis* in Response to Saline–Alkaline Stress Revealed by a Combined Proteomics and Metabolomics Method

**DOI:** 10.3390/biology13070488

**Published:** 2024-06-30

**Authors:** Tian Gao, Qiong Wang, Huarui Sun, Yang Liu, Jitao Li, Yuying He

**Affiliations:** 1 College of Fisheries and Life Science, Dalian Ocean University, Dalian 116023, China; linyigaot@163.com (T.G.); sunhuarui36@163.com (H.S.); liuyang@dlou.edu.cn (Y.L.); 2 National Key Laboratory of Mariculture Biobreeding and Sustainable Goods, Yellow Sea Fisheries Research Institute, Chinese Academy of Fishery Sciences, Qingdao 266071, China; wangqiong@ysfri.ac.cn; 3 Function Laboratory for Marine Fisheries Science and Food Production Processes, Pilot National Laboratory for Marine Science and Technology, Qingdao 266200, China

**Keywords:** *Fenneropenaeus chinensis*, proteomics, metabolomics, carbonate alkalinity stress, high pH stress

## Abstract

**Simple Summary:**

High carbonate alkalinity and high pH are the main characteristics of saline–alkaline water environments. This study aimed to elucidate the physiological mechanism of the hepatopancreas of *Fenneropenaeus chinensis* in response to saline–alkaline stress. Proteomics and metabolomics of hepatopancreas were employed to analyze the effects of high carbonate alkalinity (CA) stress and combined high carbonate alkalinity and high pH (CP) stress on shrimps after 24 h. The results indicated that several key proteins and metabolites involved in carbohydrate metabolism and fatty acid oxidation were significantly upregulated. Additionally, the antioxidant and immune systems were found to have been affected. These findings suggest that CA and CP stressors induced oxidative stress in the hepatopancreas of *F. chinensis*, resulting in impaired immunity and diminished inflammatory responses. *F. chinensis* may resist osmotic pressure imbalances caused by CA or CP stress by upregulating the energy metabolism level in the hepatopancreas. Our findings may offer valuable insights for further investigations into the saline–alkaline water culture of *F. chinensis*.

**Abstract:**

The rapid development of the mariculture industry has been hindered by limited coastal aquaculture space. To utilize the abundant inland saline–alkaline water, we studied the physiological effects of high carbonate alkalinity stress and high pH stress on *Fenneropenaeus chinensis*. The study employed quantitative proteomics by tandem mass tag (TMT) and non-targeted metabolomics analysis using a liquid chromatograph mass spectrometer (LC-MS) to understand the physiological and biochemical adaptive mechanisms of the hepatopancreas of *F. chinensis* in response to saline–alkaline stress at the molecular level. We designed two stress groups as follows: a high carbonate alkalinity (CA) group and a combined high carbonate alkalinity and high pH (CP) group. The study found that the protein and metabolic profiles of the two stress groups were changed, and the CP group, which was exposed to dual stresses, incurred more severe damage to the hepatopancreas compared to that of the CA group. After exposure to CA and CP, the hepatopancreas of *F. chinensis* showed significant alterations in 455 proteins and 50 metabolites, and 1988 proteins and 272 metabolites, respectively. In addition, *F. chinensis* upregulated the level of energy metabolism in the hepatopancreas to defend against osmotic imbalance caused by CA or CP stress, which was demonstrated by the significant upregulation of important proteins and metabolites in glycolysis, pyruvate metabolism, TCA cycle, and fatty acid oxidation. Additionally, pattern recognition receptors, the phenol oxidase system, and various immune-related metabolic enzymes and metabolites were also affected. The immune homeostasis of *F. chinensis* was affected by the alteration of the antioxidant system following exposure to CA or CP. These findings provide valuable information for *F. chinensis* saline–alkaline water cultivation practices.

## 1. Introduction

In China, there are approximately 99.13 million hectares of saline–alkaline land and 45.87 million hectares of saline–alkaline water [1]. Saline–alkaline water is distinguished by its high carbonate alkalinity, high pH, low buffering capacity, and high mineralization, which collectively limit the number of species that can inhabit these conditions [2]. Many scholars at home and abroad have recognized the importance of this problem. To develop and utilize saline–alkaline water resources, researchers utilized indigenous species that are tolerant to saline–alkaline water as animal models. By studying the physiological, biochemical, and molecular mechanisms of saline–alkaline adaptation in these fish, they were able to introduce and cultivate saline–alkaline-tolerant fish, shrimp, and crabs with stronger adaptive abilities to these types of water [3]. This approach can lead to more efficient use of these resources.

*Fenneropenaeus chinensis* is a significant economic species in China’s mariculture industry. It is native to the Yellow Sea, Bohai Sea, and other coastal areas [4]. The Chinese aquaculture industry is facing significant challenges, including coastal water pollution, excessive aquaculture densities, and frequent viral or bacterial diseases. These issues have led to higher mortality rates among *F. chinensis*, resulting in substantial economic losses. To address these challenges, inland saline–alkaline water aquaculture has the potential to alleviate the bottleneck caused by overcrowded coastal aquaculture space and insufficient production.

Carbonate alkalinity and pH are recognized as two important stressors that affect the survival, growth, and reproduction of aquatic animals in saline–alkaline waters [5]. In saline–alkaline waters, various environmental factors can interact and affect cultured animals. The high concentration of ${\mathrm{CO}}_{3}^{2-}$ and ${\mathrm{HCO}}_{3}^{-}$ in high carbonate alkaline water will irreversibly damage the respiratory system of aquatic animals, resulting in metabolic alkalosis [1]. Furthermore, the elevated pH value prevents the toxic metabolite NH_3_ from combining with H^+^, and the high concentration of NH_3_ accumulated in aquatic animals results in ammonia poisoning [6]. Lei et al. found that alkalinity and pH have an interactive effect on fish lethality. This combined effect of toxicity was also confirmed in juvenile *F. chinensis*, which showed a decrease in the survival rate with increasing alkalinity and pH [7].

The hepatopancreas, which is the largest gland in crustaceans, is responsible for secreting digestive enzymes, regulating physiological functions, defending against harmful agents, and supporting hematopoiesis. It also plays a crucial role in adapting to various adverse environments [8]. This article analyzes the molecular response mechanisms of the hepatopancreas of the *F. chinensis* under high carbonate alkalinity stress and the combined stress of high carbonate alkalinity and high pH, using a combined proteomics and metabolomics analysis. The findings will contribute to the development of saline–alkaline water culture of *F. chinensis*.

## 2. Materials and Methods

### 2.1. Animal Materials and Experimental Treatment

Healthy adult *F. chinensis* (body length = 7.35 ± 1.35 cm, body weight = 5.47 ± 2.47 g) were obtained from a shrimp farm in Rizhao, China. Before stress exposure, 350 shrimps were temporarily acclimated in an indoor aquarium for 7 days, during which the water quality was safe, with a temperature of 23~24 °C, salinity of 30 ppt, a pH of 8.2, and carbonate alkalinity of 320 mg/L as CaCO_3_. The cultural water was changed regularly every day for 1/3 of the total volume. The shrimps were fed 3.5~7% of their body weight twice a day (8:00 and 18:00) [9].

After acclimatization, 270 shrimps were randomly divided into the following three groups on average: the high carbonate alkalinity stress group (CA, carbonate alkalinity 1300 ± 40 mg/L as CaCO_3_, pH 8.2, temperature 23~24 °C, and salinity 30 ppt); the combined high carbonate alkalinity and high pH stress group (CP, carbonate alkalinity 1300 ± 40 mg/L as CaCO_3_, pH 9.0 ± 0.1, temperature 23~24 °C, and salinity 30 ppt); and the control group (CT, carbonate alkalinity 320 mg/L as CaCO_3_, pH 8.2, temperature 23~24 °C, and salinity 30 ppt). The stress experiment was conducted using 60 L aquariums with three replicates of 30 shrimps each. Water pH was adjusted using NaOH and HCl, and alkalinity was adjusted using Na_2_CO_3_, NaHCO_3_ and HCl. The stress exposure lasted for 24 h, during which the alkalinity and pH of the water were measured and corrected every 3 h using acid-base titration and a water-detecting instrument (EXO2, Yellow Spings, OH, USA, YSI). After 24 h, three shrimps were randomly selected from each aquarium of the three treatment conditions and the hepatopancreas tissue from the three shrimps was mixed in a biological replicate and stored in liquid nitrogen for proteomics analysis. At the same time, six shrimps were randomly selected from each aquarium and the above steps were repeated to form 18 samples for metabolomics analysis.

### 2.2. TMT-Labeled Quantitative Proteomics

Nine hepatopancreas samples of 50 mg each were suspended in protein lysate (8 M urea, 1% SDS, containing protease inhibitor cocktail) on dry ice and shaken three times for 40 s each time using a tissue grinder (Wonbio-96c). The mixture was lysed on ice for 30 min, followed by centrifugation at 12,000× *g* for 30 min at 4 °C to collect the supernatant. The supernatant and acetone were mixed 1:4 by vortexing (Vortex-Genie 2, SI, Austin, TX, USA) at 4 °C and the protein was precipitated overnight at −20 °C. The next day, the supernatant was removed by centrifugation at 12,000× *g* for 20 min at 4 °C. Precipitate was added to 90% precooled acetone, mixed well and centrifuged. The supernatant was discarded and the precipitate was solubilized with protein lysate (8 M urea, 1% SDS, containing protease inhibitor cocktail), sonicated on ice for 2 min, and the supernatant was collected. From each sample, 4 µL of protein was taken and extracted into clean Eppendorf tubes and then subjected to the BCA protein assay kit (Thermo Fisher Scientific, Waltham, MA, USA) according to the manufacturer’s instructions.

Subsequently, 100 μg of protein was extracted from each sample for alkylation reduction and enzymatic digestion. The samples were then labeled with TMT reagent. Liquid chromatography tandem mass spectrometry (EASY-nLC 1200 chromatography coupled with Q_Exactive HF-X mass spectrometry) was used for the two-dimensional analysis. The data were acquired using Thermo Xcalibur 4.0 software (Thermo Fisher Scientific, Waltham, MA, USA).

### 2.3. LC-MS/MS Non-Target Metabolomics

From each of the 18 samples, 50 mg of accurately weighed hepatopancreas was transferred to 2 mL centrifuge tubes. A 6 mm diameter grinding bead and 400 μL of methanol/water (4/1, *v*/*v*) extraction solvent containing 20 μg/mL internal standard (L-2-chlorophenylalanine) were added to each tube. Subsequently, the tissues were crushed using a high-throughput tissue grinder for 6 min at −10 °C, 50 Hz. Metabolites were extracted using a temperature-controlled ultrasonic cleaner (SBL-10TD, Ningbo Xinzhi Bio-technology Co., Ltd., Ningbo, China) at 5 °C, 40 Hz for 30 min. The samples were then stored at −20 °C for 30 min and centrifuged for 15 min (13,000× *g*, 4 °C). The supernatant was pipetted for LC-MS/MS analysis.

An AB SCIEX ultra-high performance liquid chromatography tandem time-of-flight mass spectrometry UHPLC-Triple TOF system was used for on-line analysis of 18 samples. The column used in the system was ACQUITY UPLC HSS T3 (100 mm × 2.1 mm i.d., 1.8 µm; Waters, Milford, CT, USA). Samples were collected using a mass spectrometer equipped with an electrospray ionization source (Triple TOF 5600+, AB SCIEX, Framingham, MA, USA) with positive and negative ion scanning modes for mass spectral signals.

### 2.4. Data Analysis

#### 2.4.1. Proteomics Data Analysis

The Proteome Discoverer software (version 2.4) was used to import the raw data for database search. Proteins meeting the criteria of Wang et al. [9] with FC > 1.20 or <0.83 and *p* value < 0.05 were considered differentially expressed proteins (DEPs). The DEPs were then annotated using the Gene Ontology (GO, http://www.geneontology.org (accessed on 17 December 2020)) and the Kyoto Encyclopedia of Genes and Genomes (KEGG, http://www.genome.jp/kegg/ (accessed on 17 December 2020)).

#### 2.4.2. Metabolomics Data Analysis

Raw data were imported into the metabolomics processing software Progenesis QI (version 2.0, WaterCorporation, Milford, CT, USA) to obtain a final data matrix. Variables with RSD ≤ 30% in QC (quality control) samples were removed. MS and MS/MS mass spectrometry information was used for matching with the metabolic databases HMDB (http://www.hmdb.ca/ (accessed on 27 November 2020)) and Metlin database (https://metlin.scripps.edu/ (accessed on 27 November 2020)). Finally, multivariate statistical analysis and screening of differential metabolites was performed using the Meggie BioCloud platform (https://www.majorbio.com (accessed on 20 December 2020)). Statistical significance was assessed using the Student’s *t*-test and *p* value. Metabolites were classified as differential metabolites if the VIP value of the metabolite was >1 and the *p* value was <0.05 [10,11].

## 3. Results

### 3.1. Proteomics Analysis

**Figure 1:**
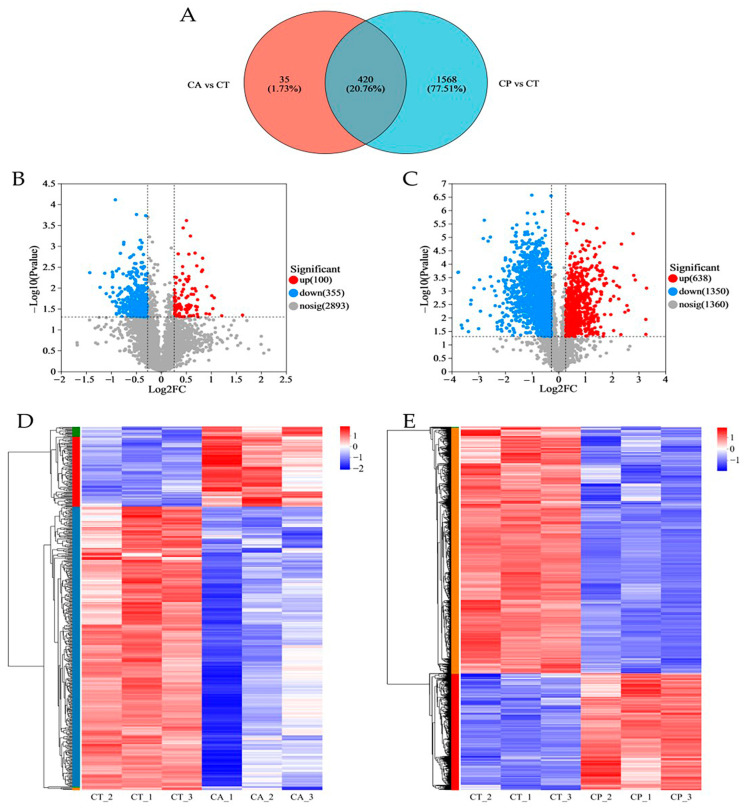
Proteomics analysis of the effects of CA and CP stress on proteins in the hepatopancreas of the *F. chinensis*: (**A**) Venn diagram of differentially expressed proteins (DEPs); (**B**) volcanic graph of DEPs in CA vs. CT group; (**C**) volcanic graph of DEPs in CP vs. CT group; (**D**) hierarchical cluster analysis of DEPs in CA vs. CT group; and (**E**) hierarchical cluster analysis of DEPs in CP vs. CT group. Red represents significantly upregulated proteins, blue represents significantly downregulated proteins, and gray indicates proteins with no significant difference in expression. The colors of the hierarchical cluster legend represent the relative expression of proteins within the group of samples.

In comparison to the CT group, the CA and CP groups had 455 and 1988 differentially expressed proteins (DEPs), respectively, with 420 common DEPs between both groups (Figure 1A–C, Table S1). Hierarchical cluster analysis of the DEPs between the CA and CP groups and the control group revealed that both CA and CP significantly affected protein expression in the hepatopancreas of *F. chinensis* (Figure 1D,E).

**Figure 2:**
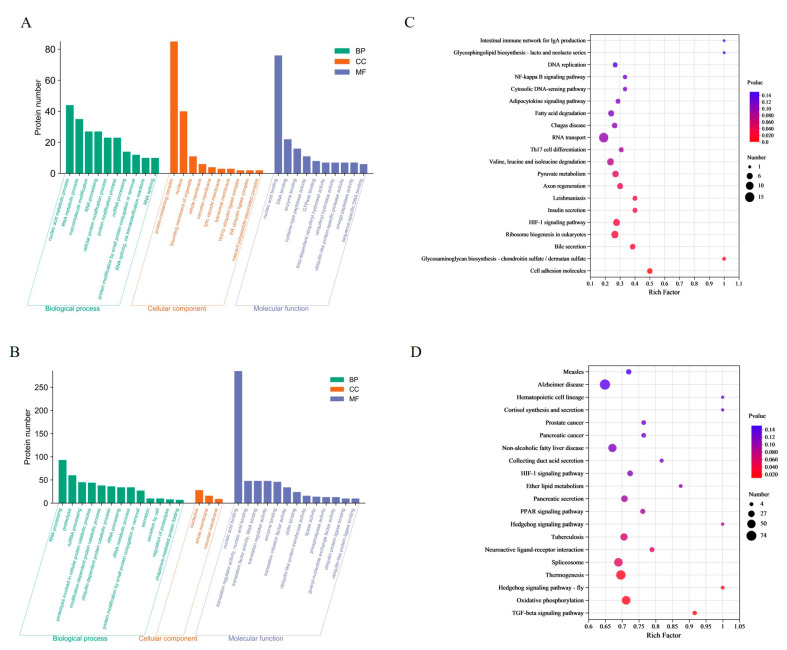
Functional enrichment analysis of DEPs: (**A**) GO analysis in CA vs. CT group; (**B**) GO analysis in CP vs. CT group. Green represents biological processes (BP); red represents cellular components (CC); and blue represents molecular functions (MF); (**C**) KEGG analysis in CA vs. CT group; and (**D**) KEGG analysis in CP vs. CT group.

In the CA group, a total of 213 DEPs were enriched to 108 terms, containing nucleic acid metabolism processes, RNA metabolism processes, protein complexes and nucleus, nucleic acid binding and DNA binding. In the CP group, a total of 499 DEPs were enriched in 29 terms. The most enriched terms were RNA processing, protein hydrolysis, nucleolus, and nucleic acid binding (Figure 2A,B). By matching the DEPs with the KEGG database, the study found that 455 DEPs from the CA group were enriched into 277 pathways (Figure 2C). Of these, nine pathways were significantly enriched for cell adhesion molecules (5 proteins), glycosaminoglycan biosynthesis-chondroitin/sulfate dermatidylin (2 proteins), and bile secretion (5 proteins). The CP group had 1988 differentially expressed proteins (DEPs) enriched in 324 pathways (Figure 2D). Four pathways showed significant enrichment: TGF-β signaling pathway (11 proteins), oxidative phosphorylation (52 proteins), hedgehog signaling pathway (7 proteins), and thermogenesis (62 proteins).

### 3.2. Metabolomics Analysis

The hepatopancreas samples from the CT, CA, and CP groups were analyzed using LC-MS. Mass spectrometry scans in both positive and negative ion modes. The samples demonstrated robust clustering in smaller areas, indicating the high stability and reproducibility of the instrument (Figure S1).

**Figure 3:**
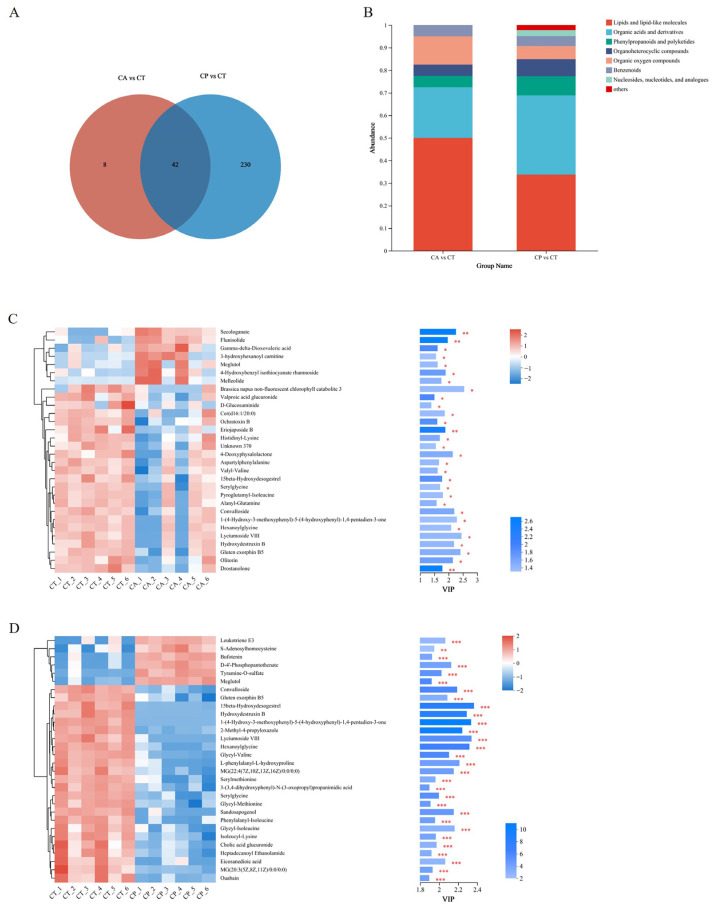
Metabolomic analysis of the effects of CA and CP stress on metabolites in the hepatopancreas of the *F. chinensis*: (**A**) Venn map; (**B**) stacked histogram of DEMs; (**C**) expression patterns and VIP values of metabolites analyzed in CA vs. CT group; and (**D**) expression patterns and VIP values of metabolites analyzed in the CP vs. CT group. Red indicates upregulation, and blue indicates downregulation. * *p* ≤ 0.05; ** *p* ≤ 0.01; *** *p* ≤ 0.001.

A total of 392 metabolites were obtained in positive (pos) mode and 424 metabolites were obtained in negative (neg) mode in the control and the two osmotic stress groups. Upon analysis, a total of 19 (pos) and 31 (neg) metabolites in the CA group showed significant changes, of which 18 were upregulated and 32 were downregulated. Similarly, in the CP group, a total of 122 (pos) and 150 (neg) metabolites underwent significant changes, of which 110 differential metabolites showed upregulation and 162 were in downregulation mode. The CA and CP groups had 42 common DEMs in comparison to the control group (Figure 3A, Table S2). Upon comparison with the HMDB 4.0 database, most of the differential metabolites in both stress groups were classified as lipid and lipid-like molecules, as well as organic acids and derivatives (Figure 3B, Table S3). The trends and expression levels of DEMs in each comparison group were visualized using clustered heatmaps and VIP bar charts, and results indicated that the hepatopancreatic metabolic profiles of *F. chinensis* in both CA and CP groups were significantly affected compared to those of the control group (Figure 3C,D).

**Figure 4:**
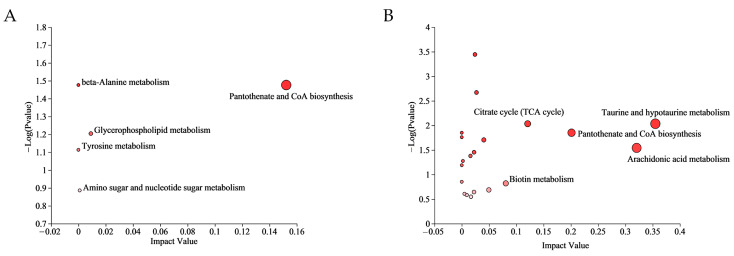
Significantly changed pathways based on the enrichment and topology analyses: (**A**) KEGG analysis in CA vs. CT group; and (**B**) KEGG analysis in the CP vs. CT group. The color of the bubbles indicates the significance of metabolite enrichment in the pathway; the size of the bubbles represents the impact value; the larger the bubble, the greater the importance of the pathway.

Multiple metabolic pathways were affected in the CA group and CP group. The differential metabolites of the CA group were enriched to 4 pathways, while the CP group had 42 enriched pathways (Table S4). The most relevant metabolic pathways in CA exposure were pantothenate and CoA biosynthesis, glycerophospholipid metabolism, amino sugar and nucleotide sugar metabolism, beta-Alanine metabolism, and Tyrosine metabolism. In addition, taurine and hypotaurine metabolism, arachidonic acid metabolism, pantothenate and CoA biosynthesis, citrate cycle (TCA cycle) and biotin metabolism were the five most representative pathways in combined CA and pH exposure (*p* value < 0.05) (Figure 4).

### 3.3. Multi-Omics Identification of Key Proteins and Metabolites

**Figure 5:**
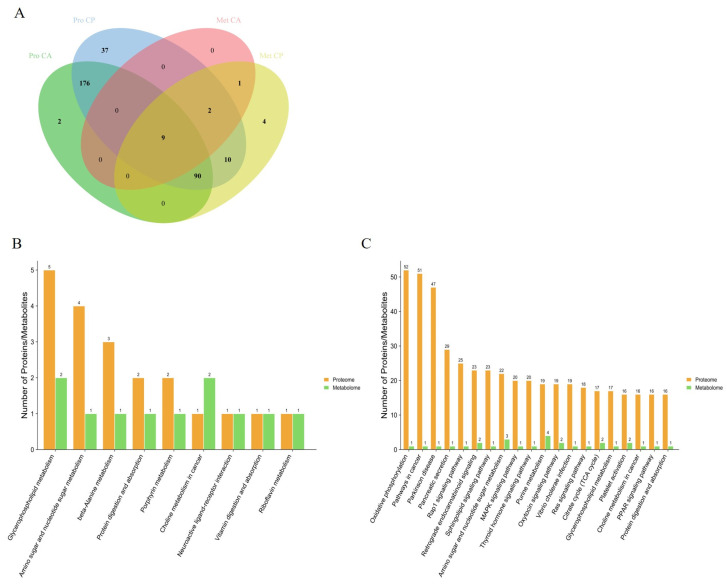
Combined analysis of KEGG pathways for DEPs and DEMs: (**A**) interactive Venn diagram of DEPs and DEMs; (**B**) KEGG pathway involving DEPs and DEMs together under CA exposure; and (**C**) KEGG pathway involving DEPs and DEMs together under CP exposure.

By comparing the pathways involved in proteins in the proteome with those involved in metabolites in the metabolome, we obtained 9 and 111 pathways common to DEPs and DEMs in the CA and CP groups, respectively (Figure 5). The pathways in which DEPs and DEMs are jointly involved in the CA group include glycerophospholipid metabolism, aminoglycan and nucleotide sugar metabolism, β-alanine metabolism, protein digestion and absorption, porphyrin metabolism, choline metabolism in cancer, neuroactive ligand–receptor interactions, vitamin digestion and absorption, and riboflavin metabolism. Notably, these nine pathways are also represented in the CP group. These nine pathways may serve as key pathways for *F. chinensis* to cope with saline–alkaline stress. In addition, the pathways co-involved in the CP group included a significant number of energy metabolism pathways, lipid metabolism pathways, signaling pathways and immune-related metabolism pathways.

**Figure 6:**
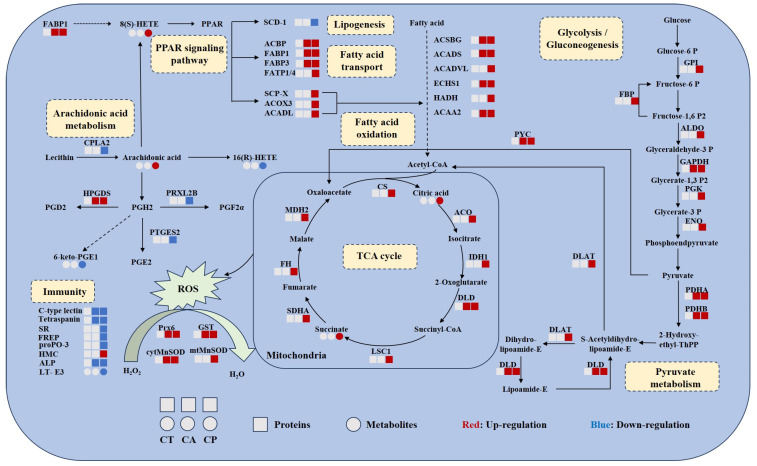
Combined proteomic and metabolomic analysis of hepatopancreas of *F. chinensis* after CA and CP stress. Circles represent metabolites and squares represent metabolites. The blue color represents downregulation and the red color represents upregulation. Solid and dashed arrows represent direct and indirect effects, respectively. FABP1, fatty acid binding protein; PPAR, peroxisome proliferator-activated receptors; scd-1, stearoyl-CoA desaturase 5-like; ACBP, acyl-CoA-binding protein; FABP3, sodium/calcium exchanger regulatory protein 1-like; FATP1/4, long-chain fatty acid transport protein 4-like; SCP-X, non-specific lipid-transfer protein, partial; ACOX3, acyl-coenzyme A oxidase 3, peroxisomal-like isoform X1; ACADL, long-chain specific acyl-CoA dehydrogenase; ACSBG, putative very-long-chain-fatty-acid—CoA ligase bubblegum isoform X2; ACADS, short-chain specific acyl-CoA dehydrogenase; ACADVL, very-long-chain specific acyl-CoA dehydrogenase; ECHS1, enoyl-CoA hydratase; HADH, hydroxyacyl-coenzyme A dehydrogenase; ACAA2, 3-ketoacyl-CoA thiolase; GPI, glucose-6-phosphate isomerase-like; FBP, fructose-1,6-bisphosphatase 1-like isoform X1; ALDO, fructose 1,6-biphosphate-aldolase A; GAPDH, glyceraldehyde-3-phosphate dehydrogenase isoform X1; PGK, phosphoglycerate kinase; ENO, phosphopyruvate hydratase; PDHA, probable pyruvate dehydrogenase E1 component subunit alpha; PDHB, pyruvate dehydrogenase E1 component subunit beta; DLD, dihydrolipoyl dehydrogenase; DLAT, dihydrolipoyllysine-residue acetyltransferase component of pyruvate dehydrogenase complex; PYC, pyruvate carboxylase; CS, probable citrate synthase 1; ACO, aconitate hydratase; IDH1, isocitrate dehydrogenase; LSC1, succinate—CoA ligase subunit alpha; SDHA, succinate dehydrogenase [ubiquinone] flavoprotein subunit; FH, fumarate hydratase; MDH2, malate dehydrogenase; CPLA2, cytosolic phospholipase A2-like isoform X2; HPGDS, hematopoietic prostaglandin D synthase; PRXL2B, prostamide/prostaglandin F synthase-like; PTGES2, prostaglandin E synthase 2; SR, scavenger receptor; FREP, fibrinogen-related protein; ProPO-3, prophenoloxidase-3; HMC, hemocyanin; ALP, alkaline phosphatase; T-E3, leukotriene E3; Prx6, peroxiredoxins; GST, glutathione S-transferase; cytMnSOD, cytoplasmic manganese superoxide dismutase; mtMnSOD, mitochondrial manganese superoxide dismutase.

The relationships between DEPs and DEMs in key metabolic pathways were summarized, and the results suggest that both CA and CP affect the protein-metabolite network in the hepatopancreas of *F. chinensis* (Figure 6). DEPs and DEMs associated with glycolysis/gluconeogenesis, fatty acid oxidation, TCA cycle, pyruvate metabolism, and PPAR signaling pathway were upregulated, indicating increased energy production. The antioxidant enzyme system was activated, indicating that exposure to CA and CP caused oxidative stress in *F. chinensis*. Meanwhile, arachidonic acid metabolism and immune response-related DEPs and DEMs were downregulated, indicating that the immune system of *F. chinensis* was suppressed.

## 4. Discussion

In recent years, a growing number of studies have used omics approaches to explore the processes by which saline–alkaline-tolerant aquatic organisms adapt to complex and changing environments. Integrated proteomics and metabolomics investigations can better explain the molecular mechanisms of diseases or external stimuli, as well as the regulatory mechanisms of various groups on pathways [12]. In this study, we integrated proteomics and metabolomics analyses to compare the differences among CA, CP, and CT groups. We found that in proteomics, the number of downregulated DEPs was much greater than the number of upregulated proteins in both the CA group and CP group. In addition, DEPs were more abundant in the CP group than in the CA group. This result was also in accordance with the metabolomics results. This suggests that short-term exposure to CA and CP resulted in dramatic changes in the expression levels of hepatopancreatic proteins and metabolites in *F. chinensis*. Under dual exposure to the environmental stress of high carbonate alkalinity and high pH, *F. chinensis* responded to the stress more drastically than to a single stress, which may be due to the synergistic toxic effects of carbonate alkalinity and pH [13]. In order to withstand greater environmental stress, *F. chinensis* maintains the balance of the organism’s internal environment by mobilizing the expression of more proteins and metabolites.

### 4.1. Enhanced Carbohydrate and Energy Metabolism

As is well known, it is essential for aquatic organisms to possess effective ionic and osmoregulatory systems in order to adapt to a variety of aquatic settings [14]. Nevertheless, in order to maintain osmotic pressure stability, it may be necessary to consume a greater quantity of energy, and carbohydrate metabolism represents a critical source of energy for ionic and osmotic control [15,16]. 

Pyruvate metabolism is a major pathway of carbohydrate metabolism, and it plays an important regulatory role in the process of glucose oxidation and fatty acid metabolism. In this study, significant upregulation of pyruvate-related proteins was observed in the CA and CP groups, indicating altered hepatopancreatic metabolism and increased energy production in *F. chinensis* under saline–alkaline exposure. The TCA cycle serves as a hub, linking carbohydrate, fatty acid, and amino acid metabolism, and its intermediates can also act as signaling molecules that drive a variety of cellular functions [17,18]. In the CP group, TCA-cycle-associated metabolites and proteins were significantly upregulated. Moreover, several glycolysis-related proteins were significantly increased by the CP group. (Figure 6). It is suggested that *F. chinensis* resist the high osmotic environmental stress by accelerating the glycolysis and TCA cycle to provide more energy to the organism and produce antioxidants when faced with the double stress of pH and carbonate alkalinity [19]. Oxidative phosphorylation is a key process for ATP production in mitochondria. Reducing equivalents (NADH and FADH2) generated during glycolysis and the TCA cycle sequentially convert ADP to ATP via the electron transport chain [20,21]. Oxidative phosphorylation has been shown to be highly involved in marine organisms in response to environmental stress [22,23]. In this study, a total of 52 DEPs associated with the oxidative phosphorylation pathway were identified in the CP group; 38 DEPs were significantly upregulated; and 14 DEPs were downregulated (Table S5). Ren et al. found that oxidative phosphorylation processes were disrupted in *Panulirus ornatus* in response to environmental stress [23]. These results suggested that oxidative phosphorylation plays an important regulatory role in response to stress in aquatic animals.

### 4.2. Oxidative Stress Occurred under CA and CP Stress

Oxidative stress is a state in which there is an imbalance between oxidative and antioxidant effects in organisms [24]. It is related to the accumulation of excessive reactive oxygen species (ROS) in organisms. Previous studies have demonstrated that carbonate alkalinity and pH stressors cause an excess of ROS to be produced, which in turn triggers the activation of antioxidant systems in crustaceans [25,26].

Peroxiredoxins (*Prxs*) constitute a family of cysteine-based peroxidases with the capacity to reduce a diverse array of inorganic and organic peroxides. Consequently, they play a pivotal role in regulating intracellular ROS levels [27,28]. Our observations indicate that *Prx6* was significantly upregulated in both the CA and CP groups. The results of our study confirmed the protective role of *Prx6* in *F. chinensis* against oxidative stress induced by CA and CP. Manganese superoxide dismutase (*MnSOD*) is a major scavenger of harmful ROS metabolites in the mitochondrial matrix [29,30]. Our proteomic data showed a significant increase in *cytMnSOD* content in the CA group and a significant upregulation of both *cytMnSOD* and *mtMnSOD* in the CP group compared to the CT group. This is consistent with the high expression of *mtMnSOD* in the hepatopancreas of *Litopenaeus vannamei* under high-temperature and hypoxic conditions [31]. 

In addition, a significantly elevated expression of 4-hydroxynonenal (*HNE*) was found in the metabolomics data of the CP group. *HNE* is a highly reactive lipid peroxidation product of oxidative stress and can be regarded as a biomarker of oxidative stress [32,33]. Interestingly, glutathione S-transferase (*GST*) has been shown to detoxify *HNE* in the liver of largemouth bass (*Micropterus salmoides*) [34]. However, we found that *GST* was significantly upregulated in the CP group, while *HNE* expression was also increased. This might be explained by the fact that the combined stress environment puts more strain on *F. chinensis* to survive, which causes ROS levels to skyrocket. This, in turn, results in a lack of timely scavenging, which causes polyunsaturated fatty acids on the cell membrane to undergo rapid lipid peroxidation with free radicals, which in turn produces lipid peroxidation products like HNE. The above results indicated that oxidative stress occurred in medium-salt berries in both the CA and CP groups under carbonate alkalinity and pH conditions. Furthermore, the dual environmental stresses might aggravate oxidative stress.

### 4.3. CA and CP Stress Caused Lipid Metabolism Disorders

Lipids are essential for many functions in organisms. They serve as messenger molecules, produce and store energy, actively participate in membrane function, and have critical immunological roles [35,36,37]. Several studies have shown that saline–alkaline exposure leads to abnormal lipid metabolism in aquatic animals [38,39,40,41]. In our study, we discovered that the hepatopancreas of the *F. chinensis*, had a strong lipid metabolic response to acute changes in carbonate alkalinity and pH (Figure 6). During the analysis of DEMs, it was discovered that the glycerophospholipid metabolic pathways were altered in the CA group, while the arachidonic acid metabolism and PPAR signaling pathways were significantly enriched in the CP group, according to the KEGG pathway analysis.

Phosphatidylcholine and phosphatidylethanolamine are essential for maintaining cell membrane permeability and stability [42]. Glycerophospholipid metabolism is also a significant pathway involved in systemic immunity and low-grade inflammatory states [43]. Abiotic stress-induced ROS can attack membrane lipids, leading to lipid peroxidation. This affects the normal physiological function of cell membranes and can result in inflammatory damage due to physiological imbalances in the organism [26]. The study detected an increase in lysophosphatidylcholine (LysoPC (22:1(13Z)), LysoPC (20:1(11Z))) lysophosphatidylethanolamine (LysoPE (15:0/0:0)) and phosphatidylethanolamine PE (15:0/20:0)) content in *F. chinensis* after hypercapnic alkalinity stress, indicating that the shrimps synthesized more glycerophospholipids to maintain cell membrane stability and resist inflammation. This result was also observed in the combined high carbonate alkalinity and high pH stress group.

Arachidonic acid is a polyunsaturated fatty acid produced by phosphatidylcholine in the presence of cell membrane phospholipases [44]. As an intracellular signaling molecule, it increases resilience to stress by preventing adipocyte proliferation and apoptosis, and lowering chemokines linked to inflammation [45]. The metabolomics study of the CP group revealed increased levels of arachidonic acid, 11β-prostaglandin E2 (*11β-PGE2*), leukotriene E3 (*LT-E3*), and 8(S)-HETE, and decreased levels of 16(R)-HETE and 6-keto PGE1. These findings suggest that the dual stresses of high carbonate alkalinity and high pH cause lipid metabolism disorders in *F. chinensis*, resulting in inflammatory responses.

### 4.4. CA and CP Stress Triggered Immune Response

*F. chinensis* is an invertebrate that relies primarily on innate immunity to protect itself from microbial infections [46]. It is generally accepted that shrimps recognize general pathogen-associated molecules and activate immune responses mainly through pattern recognition receptors (*PRRs*) [47]. In this study, several *PRRs* (C-type lectin, tetraspanin, scavenger receptor (*SR*) and fibrinogen-related protein (*FREP*)) were induced to be significantly downregulated in the CP group. This finding is consistent with that of with previous studies, in which a decrease in the expression of C-type lectin in the protein profile of *F. chinensis* under high pH [48] and low pH [17] conditions was observed. 

Prophenoloxidase (*proPO*) activation is important in the development of the immune response in shrimps [49]. In the present study, the expression of *proPO-3* was downregulated in *F. chinensis* under CP stress, probably due to the inability of the antioxidant system to scavenge excessive free radicals and damage to hepatopancreatic tissues, resulting in a decrease in *proPO-3* activity. Alkaline phosphatase (*ALP*) is a hydrolyzing enzyme that can be utilized as an immunoenzyme to assess specific indicators of immune function and health in crustaceans [46]. Our findings indicate that *ALP* expression was downregulated to varying degrees in the CA and CP groups. Numerous studies have shown that high carbonate alkalinity and pH stress can cause damage to the hepatopancreatic structure of crustaceans [50,51]. In a recent study, Zhao et al. found that T-2 toxin can induce hepatopancreatic damage and significantly reduce *ALP* activity in the *Eriocheir sinensis* [52]. Therefore, it is possible that excessive environmental stress in the CA and CP groups caused significant damage to the hepatopancreatic structure of *F. chinensis*, which may have inhibited *ALP* activity.

Furthermore, the KEGG analysis indicated significant alterations in immune-related metabolic pathways in both the CA and CP groups. The proteomics results revealed a significant enrichment of cell adhesion molecules and leishmaniasis pathways under CA exposure. Under CP stress, the TGF-β signaling pathway was enriched in proteomics, along with endocrine resistance, amoebiasis, leukocyte transendothelial migration, and inflammatory mediator regulation of TRP channels in metabolomics, all highly correlated with immune defense. This suggests that exposure to high levels of carbonate alkalinity, as well as exposure to both high carbonate alkalinity and high pH, have different effects on the immune system of *F. chinensis*. These effects may be modulated through different pathways in response to environmental changes.

## 5. Conclusions

In summary, this study investigated the metabolic and molecular responses of the hepatopancreas of the *F. chinensis* to CA and CP stress using TMT-labeled quantitative proteomics and LC-MS non-targeted metabolomics analysis. The study demonstrated that both CA and CP stress impacted the protein and metabolic profiles in the hepatopancreas of *F. chinensis*. The number and expression of DEPs and DEMs in response to dual environmental stress were generally higher in the CP group than in the CA group stressed by a single environmental factor. CA and CP stress induced oxidative stress in the hepatopancreas of *F. chinensis*, resulting in lipid peroxidation and disruption of lipid metabolism, and further leading to decreased immunity in *F. chinensis*, triggering an inflammatory response and damaging the immune system. Under both CA and CP stress, *F. chinensis* resist osmotic environments by increasing energy synthesis through enhanced carbohydrate metabolism and other pathways. These results provide insights into the mechanisms of tolerance to CA and CP stress in the hepatopancreas of *F. chinensis* and will provide a theoretical basis for further saline–alkaline water cultivation of *F. chinensis*.

## Data Availability

Proteomics sequencing and metabolomics sequencing data from this study will be made available upon request.

## Supplementary Materials

The following supporting information can be downloaded at https://www.mdpi.com/article/10.3390/biology13070488/s1, Figure S1: Quality analysis of metabolomics data. Table S1: Analysis of significantly differentially expressed proteins. Table S2: Analysis of significantly differentially expressed metabolites. Table S3: Classification of differential metabolites. Table S4: KEGG pathway enrichment statistics for metabolomics DEMs. Table S5: DEPs in the oxidative phosphorylation pathway in the CP group.
