# Supplementary material for: Physiological Adaptation of Fenneropenaeus chinensis in Response to Saline–Alkaline Stress Revealed by a Combined Proteomics and Metabolomics Method
Source: Biology (Basel). 2024 Jun 30;13(7):488. doi: 10.3390/biology13070488 (PMC11274245; doi:10.3390/biology13070488)
Supplement: Supplementary file 1 [file biology-13-00488-s001.zip › Figure S1. Quality analysis of metabolomics data.pdf]

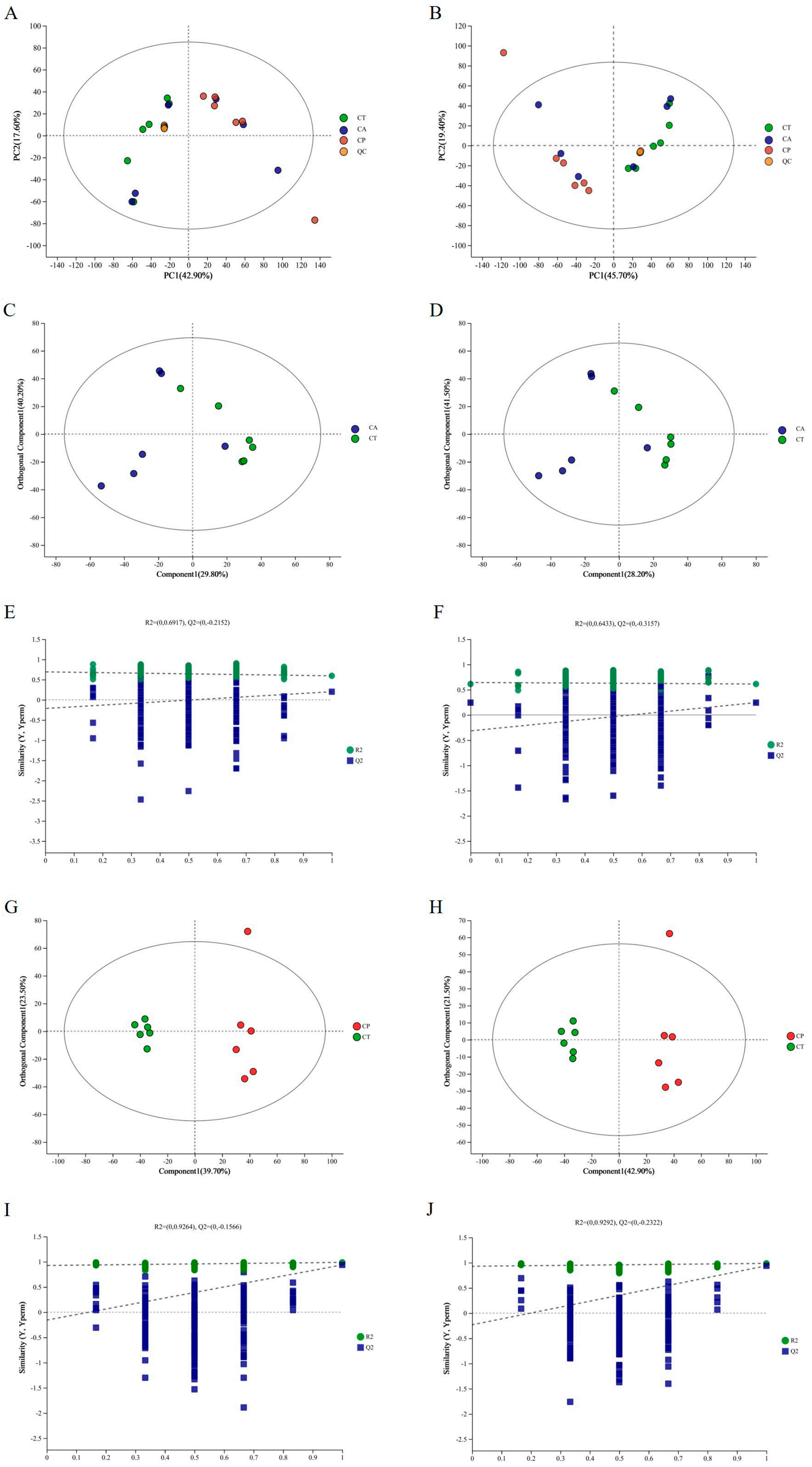

A
PC2(17.60%)
PC1(42.90%)
CT
CA
CP
QC
B
PC2(19.40%)
PC1(45.70%)
CT
CA
CP
QC
C
Orthogonal Component1(40.20%)
Component1(29.80%)
CA
CT
D
Orthogonal Component1(41.50%)
Component1(28.20%)
CA
CT
E
R2=(0,0.6917), Q2=(0,-0.2152)
Similarity (Y, Yperm)
R2
Q2
F
R2=(0,0.6433), Q2=(0,-0.3157)
Similarity (Y, Yperm)
R2
Q2
G
Orthogonal Component1(23.50%)
Component1(39.70%)
CP
CT
H
Orthogonal Component1(21.50%)
Component1(42.90%)
CP
CT
I
R2=(0,0.9264), Q2=(0,-0.1566)
Similarity (Y, Yperm)
R2
Q2
J
R2=(0,0.9292), Q2=(0,-0.2322)
Similarity (Y, Yperm)
R2
Q2

Figure S1. Quality analysis of metabolomics data. (A) PCA analysis in POS mode. (B) PCA analysis under NEG mode. (C) OPLS-DA analysis in POS mode under CA stress. (D) OPLS-DA analysis in NEG mode under CA stress. (E) OPLS-DA alignment test in POS mode under CA stress. (F) OPLS-DA alignment test in NEG mode under CA stress. (G) OPLS-DA analysis in POS mode under CP stress. (H) OPLS-DA analysis in NEG mode under CP stress. (I) OPLS-DA alignment test in POS mode under CP stress. (J) OPLS-DA alignment test in NEG mode under CP stress.
